# Losing ground: projections of climate-driven bloom shifts and their implications for the future of California’s almond orchards

**DOI:** 10.1038/s41598-023-50688-y

**Published:** 2024-01-05

**Authors:** Jessica Orozco, Oren Lauterman, Or Sperling, Tarin Paz-Kagan, Maciej A. Zwieniecki

**Affiliations:** 1 Plant Sciences, UC Davis, Davis, CA USA; 2 Department of Mapping and Geoinformation Engineering, Civil and Environmental Engineering, Technion-Israel Institute of Technology, Haifa, Israel; 3 Plant Sciences, ARO-Volcani, Rishon LeZion, Israel; 4 French Associates Institute for Agriculture and Biotechnology of Dryland, The Jacob Blaustein Institutes for Desert Research, Ben-Gurion University of the Negev, Sede Boqer Campus, 8499000 Beersheba, Israel

**Keywords:** Environmental impact, Ecophysiology

## Abstract

Climate change is expected to impact the spring phenology of perennial trees, potentially altering the suitability of land for their cultivation. In this study, we investigate the effects of climate change on the bloom timing of almond orchards, focusing on California, the world's leading region for almond production. By analyzing historical climatic data, employing a model that considers hourly temperatures and fall non-structural carbohydrates to predict bloom dates, and examining various Coupled Model Intercomparison Project Phase 6 (CMIP6) scenarios, we assess the potential impacts of climate shifts on plant phenology and, consequently, on land suitability for almond farming. Our findings reveal that, within the next 30 years, the land suitable for almond production will not undergo significant changes. However, under unchanged emission scenarios, the available land to support almond orchard farming could decline between 48 to 73% by the end of the century. This reduction corresponds with an early shift in bloom time from the average Day of Year (DOY) 64 observed over the past 40 years to a projected earlier bloom between DOY 28–33 by 2100. These results emphasize the critical role climate shifts have in shaping future land use strategies for almond production in Central Valley, California. Consequently, understanding and addressing these factors is essential for the sustainable management and preservation of agricultural land, ensuring long-term food security and economic stability in the face of a rapidly changing climate.

## Introduction

Climatic conditions can significantly impact the spring phenology of woody perennials and consequently strongly influence species physiology, fecundity, competitiveness, and survival. An iconic example of the influence of climate on spring bloom is observed in the annual cherry blossom event in Kyoto City, where shifts toward earlier blooms, attributed to global warming have been recorded^1^. This example isn’t isolated; recent phenological research consistently highlights the pervasive influence of global warming on bloom timing^2,3^. However, the effects of climate change aren’t limited to changes in bloom timing alone; they also have substantial implications for bloom synchronicity^3–5^. For species that rely on synchronized blooming, particularly dioecious and self-incompatible ones, this synchronicity is essential for reproductive success, aligning with heightened pollinator activity and bolstering horticultural productivity. These alterations in bloom dynamics can have upstream repercussions on land suitability, potentially enhancing or restricting the areas conducive to species survival and for horticultural contexts, their optimal productivity. Therefore, it is essential to examine the potential impacts of climate change on the spring phenology of long-lived, high-input cropping systems in the upcoming years. Predicting climate’s effect on bloom time will help address uncertainties associated with new orchard plantation establishment and ensure the sustainability of these cropping systems.

The Central Valley in California, USA, is a dominant force in global almond production, producing 80% of the world's almond supply. In the US, California’s almond industry holds the distinction of being the top crop export by value. Furthermore, within the state of California, almonds claim the largest dedicated cultivation area compared to any other crop, an area that continues to expand annually^6^. Therefore, it stands as a poignant case study to understand these climate-driven phenological shifts and their consequent impacts on land suitability. Almond phenology is intrinsically linked to climate factors; an early bloom can compromise fruit set due to potential late frost exposure, while a late bloom can face pollen inactivity due to high temperatures. Notably, the climate, particularly temperatures experienced during dormancy, exerts a significant influence on spring phenology. Specifically, an accumulation of chill hours is necessary to initiate dormancy break in response to spring warming^7,8^. Multiple statistical approaches have harnessed the strong correlation between bloom and the accumulation of chill and heat hours to develop phenological models. Still, in most cases, these empirical methods can only determine the readiness of trees to bloom, not the exact date^4,9^. Furthermore, while it is widely recognized that winter temperatures play a crucial role in determining the bloom timing of temperate perennials, the mechanisms by which trees chronicle and integrate temperature information remain unclear. Many existing bloom models rely on empirically-based algorithms and, unfortunately, do not account for underlying physiological processes. Addressing this gap, the C-T model^10,11^ was developed to predict bloom dates, achieving an accuracy within a root mean square error (RMSE) of 4.7 days^10^, by integrating ambient temperatures with carbohydrate-dependent metabolic kinetics. It rests upon the concept that deciduous trees dynamically chronicle winter temperatures through two responsive physiological processes tied to temperature fluctuations^12^, starch synthesis and starch digestion, to delve into the historical and prospective impact of winter temperatures on almond bloom timing in the Central Valley, CA and to understand the subsequent implications on land suitability for almond orchards. For this future prospective, we draw upon the Coupled Model Intercomparison Project Phase 6 (CMIP6) state-of-the-art projections which provide insights into the possible climate futures by varying greenhouse gas concentration trajectories^13^. By analyzing CMIP6 scenarios, we seek to understand the future climatic impacts on almond phenology and forecast the future land suitability of the California Central Valley for sustained almond production in light of anticipated climatic changes.

## Materials and methods

### Historic climate trends

First, we needed to determine the spatial and temporal variability in California’s Central Valley winter temperatures. We used PRISM gridded four-by-four km daily minimum and maximum temperatures (T_PRISM max|min_) for Central Valley, California, focusing on winters ranging from October 15th to April 30th from the fall of 1981 till the spring of 2022 (PRISM Climate Group, Oregon State University, https://prism.oregonstate.edu). Past winter average maximum and minimum temperatures were calculated for a period as:

1 $${\overline{{\text{T}}} }_{{\text{PRISM\, max}}|\mathrm{ min}}=\frac{\sum_{{15}{\text{th\, October}}({\text{year}})}^{{30}{\text{th\, April}}({\text{year}}+1)} \frac{\sum {\mathrm{ T}}_{\mathrm{PRISM\, max}|{\text{min}}}}{\mathrm{number\, of\, grid\, points}}}{\mathrm{number\, of\, days}}$$

### Carbohydrate-temperature bloom model (C–T model)

Here we apply the C–T model to predict blooms times across the Central Valley, California at a resolution of four-by-four km. For full model development and description please refer to Sperling et al. 2019^10^ and 2021^11^. Briefly, the model posits that dormant trees adjust their starch metabolism to maintain a metabolic soluble sugar homeostasis throughout winter by modifying their starch degradation or synthesis pathways. Since these metabolic processes are closely tied to temperature, the C–T model integrates hourly temperatures. These metabolic changes influence soluble carbohydrates (SC) dynamics ultimately determining when SC concentrations hit a threshold that triggers blooming.

Metabolic activity rates in relation to temperature [K(T)] are characterized by exponential curves defined by a frequency factor (α) and the specific energy of activation (β; Eq. 2).

2 $$K\left(T\right)=\alpha \cdot {\text{exp}}(\beta \cdot T)$$

The C–T model^10,11^ links these processes with the regulation of starch and soluble sugar pools ([St] and [SC], respectively) by the activity of starch synthesis [Ks(T)] and starch degradation [Kd(T)] in consideration of cellular respiration [R(T); Eq. 3].

3 $$St\frac{Kd(T)\to }{\leftarrow Ks(T)}SC-R(T)$$

The model was parametrized and tested on bloom data from four locations in the CA Central Valley and winter hourly temperatures for 35 consecutive years (1982–2017)^10^ an initiated on an October average of SC content retrieved from the Carbohydrate Observatory. The C–T model requires initial levels of starch and sugar content along with hourly winter temperatures. Starch and sugar data were sourced from the Carbohydrate Observatory (https://zlab-carb-observatory.herokuapp.com/), a long-term study which gathers NSC data from across the Central Valley, CA for multiple species^14^. Furthermore, given that NSC data encompasses the Central Valley, latitudinal estimates for each sugar and starch were generated to account for latitudinal differences. Hourly winter temperatures were generated from daily maximum and minimum values following the approach outlined by Sperling and Zwieniecki^11^.

### Historic and future bloom projections

We generated forecasts for the Day of the Year (DOY) representing the bloom date within each 4 × 4 km grid across the Central Valley, over a span of 40 years (1982–2022).

To predict the influence of climate change on winter temperatures for the years 2050 and 2100, we utilized two CMIP6 scenarios, SSP5-8.5 (no emission control) and SSP1-2.6 (emission reduction)^13^. This involved adjusting winter minimum and maximum temperatures according to the projected changes in CMIP6 surface air temperatures (ΔT_SSP_). Specifically, the SSP5-8.5 scenario predicts an increase of 1.6 °C and 3.8 °C for 2050 and 2100 respectively, while SSP1-2.6 anticipates a rise of 1.1 °C for both time points (Eq. 4; scenarios: SSP5-8.5ΔT_min•max_, SSP1-2.6 ΔT_min•max_). Additionally, we added a hypothetical condition where only the maximum temperature increases while the minimum temperature remains constant (as per Eq. 5; scenarios: SSP5-8.5ΔT_max_, SSP1-2.6ΔT_max_). Moreover, to account for interannual winter temperature and geographical distribution variability, T_PRISM_ data from each of the previous four years (2018, 2019, 2020 and 2021) was used and for each grid point new daily min and max were calculated as:

4 $${{\text{T}}}_{{{\rm FUTURE\, min} \cdot {\rm max}}}={{\text{T}}}_{\mathrm{PRISM\, max}+\Delta {{\text{T}}}_{\mathrm{SSP }}|{{\min}}+\Delta {{\text{T}}}_{{\text{SSP}}}}$$

5 $${{\text{T}}}_{\mathrm{FUTURE\, max}}={{\text{T}}}_{\mathrm{PRISM\, max}+2\Delta {{\text{T}}}_{\mathrm{SSP}}|{\min}}$$

### Comparing historical bloom predictions with future bloom projections

To assess how the different scenarios (SSP5-8.5ΔT_min • max_, SSP1-2.6ΔT_min • max_, SSP5-8.5ΔT_max_, SSP1-2.6ΔT_max_) would impact bloom time in comparison to the past (1982–2022) using R Statistical Software (v4.2.2: R Core Team 2022) we constructed a linear regression model where each scenario served as a predictor for bloom date (DOY). This was followed by executing an Analysis of Variance (ANOVA) paired with a post-hoc Dunnett’s test contrast analysis.

## Results

**Figure 1:**
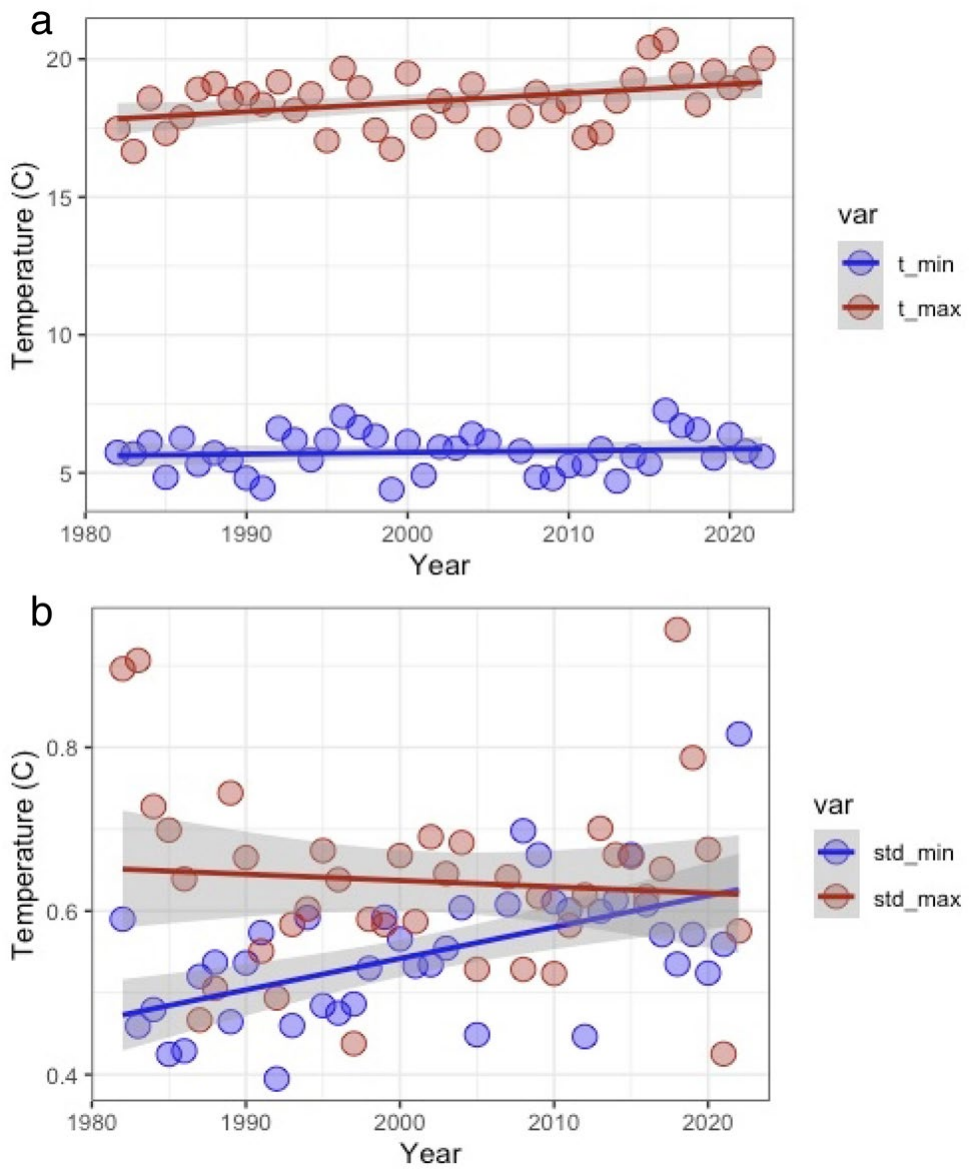
Historic climate conditions. (**a**) Average daily maximum temperature (t_max) and average daily minimum temperature (t_min) in Central Valley, CA, USA, between 1982 and 2022. There was a significant upward trend of t_max but not in t_min. (**b**) Change in the standard deviations of t_max and t_min in Central Valley, CA, USA, between 1982 and 2022. There was a significant upward trend of t_min std but not in t_min.

For the last 40 years, we observed a significant cumulative increase of 1.4 °C (~ 0.033 °C year^−1^; p = 0.00932; Fig. 1) in winter (October 15th–April 30th) mean maximum temperatures, from 17.8 to 19.2 °C. The standard deviation of maximum mean temperature across the Central Valley remained constant at ~ 0.06 °C (Fig. 1b) for 40 years. Mean minimum winter temperature remained almost constant during the last 40-year period, changing only by 0.3 °C from 5.6 to 5.9 °C (0.006 °C year^−1^; p = 0.532; Fig. 1a). Interestingly, there was a significant increase in standard deviation from 0.46 to 0.63 °C (p = 0.000189; Fig. 1b) of the mean minimum temperature suggesting higher nightly variability.

**Figure 2:**
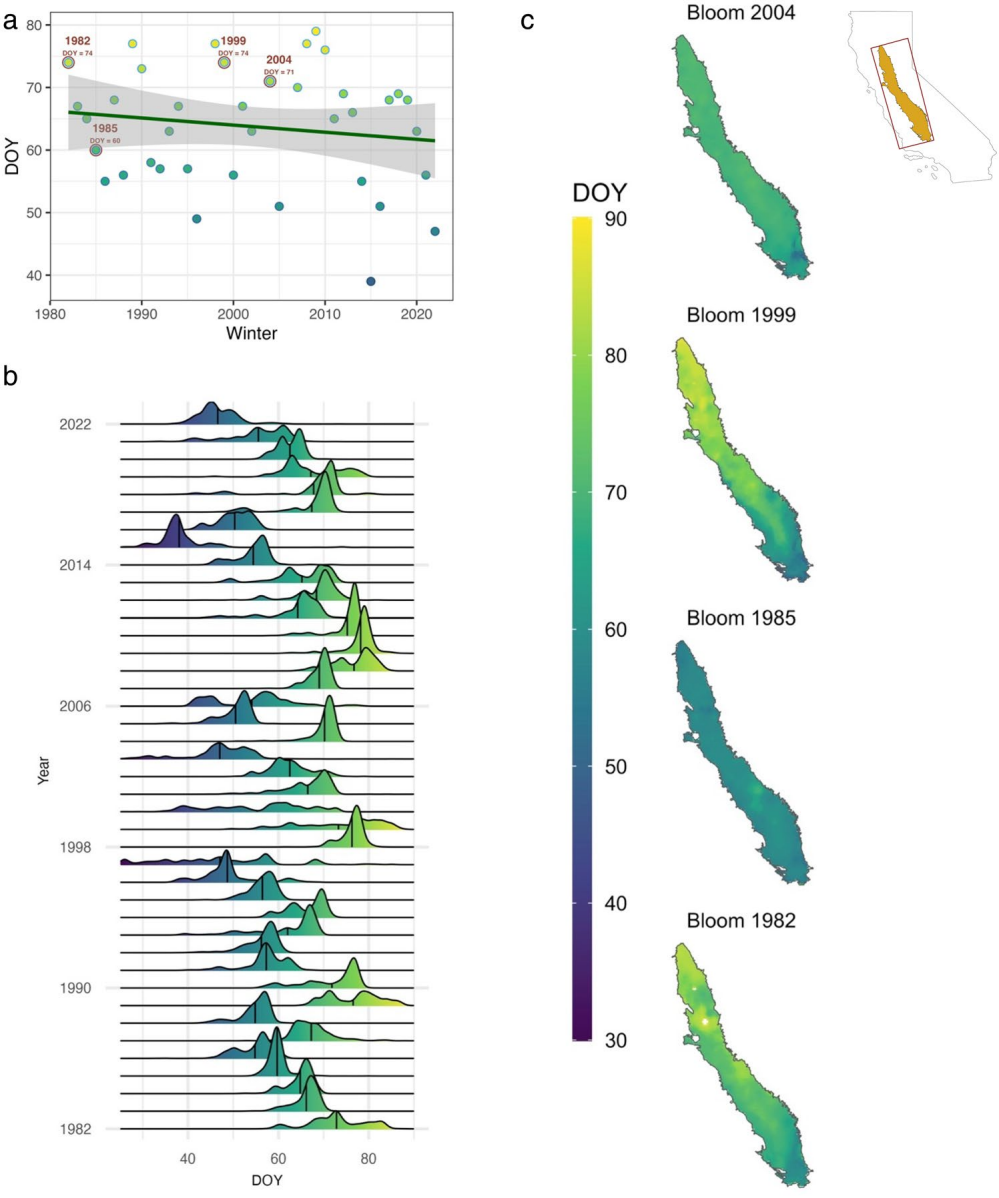
Historic bloom time projections. (**a**) Modeled average historic bloom day of the year (DOY) for Central Valley, CA. (**b**) Density plot of bloom DOY across California’s almond growing regions. (**c**) Four examples of bloom DOY across Central Valley, CA, depicting synchronous (1985 and 2004) and asynchronous (1982 and 1999) bloom patterns.

Our projections of almond bloom dates across California’s Central Valley, based on the data from the past 40 years (1982–2022), demonstrated significant variations in bloom dates. On average, the predicted bloom day advanced by 6.5 days in 40 years, shifting from March 7th to March 2nd (on average, trees bloomed on March 5th). The bloom delay corresponds to a change in DOY from 66 to 61 and an average bloom at the 64th DOY. The inter-annual variability in peak bloom, when 70% of blossoms have opened, ranges from February 5th to March 16th (35th to the 75th DOY; Fig. 2a). There is also an annual variability in how bloom is synchronized across California’s Central Valley. For instance, in 1985 and 2004, the bloom exhibited a single peak time (DOY 60 and 71, respectively) and spanned ten days across the entire Central Valley, indicating strong synchronicity. On the other hand, in 1982 and 1999, the bloom prediction extended over 25 days without a discernible peak (both DOY 74), demonstrating a high degree of asynchrony (Fig. 2b). In such asynchronous years, there was a solid latitudinal gradient in bloom time, with regional differences between the north and south (Fig. 2c).

**Figure 3:**
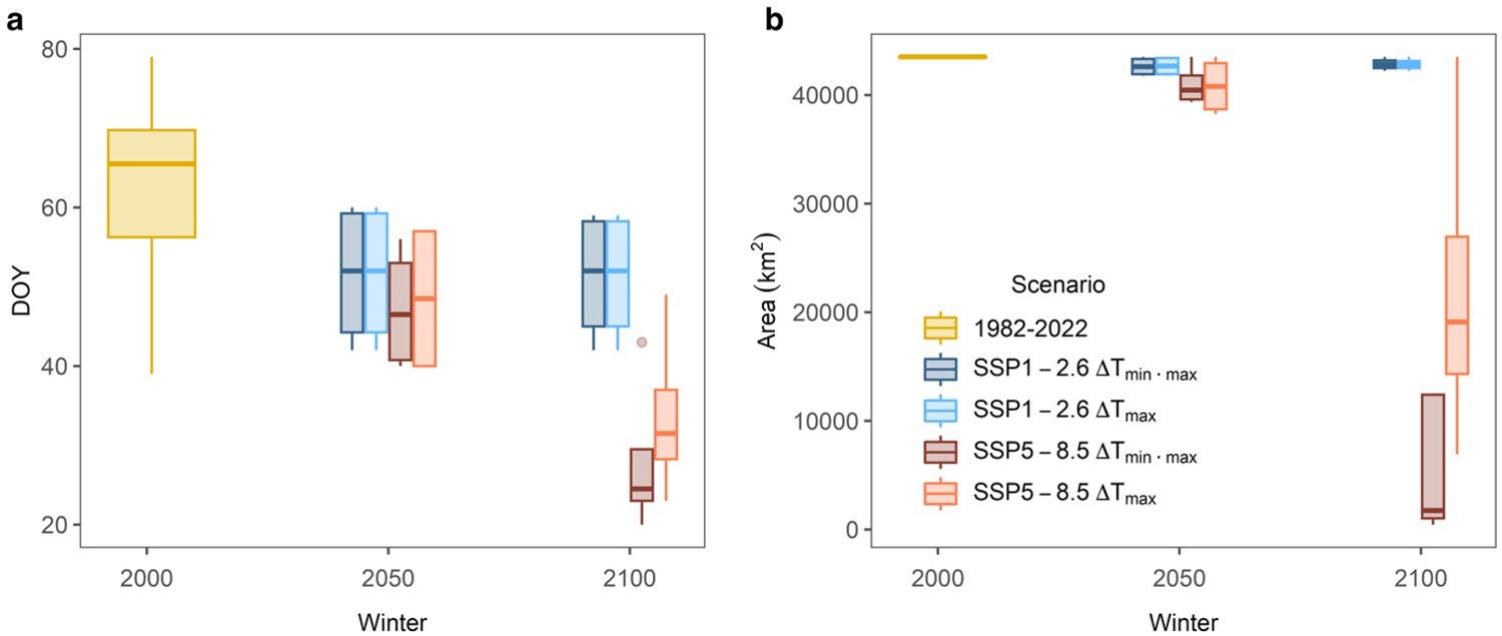
Projected bloom dates and almond-supporting areas. (**a**) Projected bloom day of year (DOY) highlighting historical data (1982–2022) and under various climate change scenarios in 2050 and 2100. (**b**) Total areas in the Central Valley, CA supporting bloom for each scenario constrained within February 1st (DOY 32) and March 31st (DOY 90). Boxplots showcase the interquartile ranges for both bloom dates and almond production areas.

We utilized the projected average global temperature increases for 2050 and 2100 from the CMIP6 climate scenarios to assess the effects of future climate shifts on almond bloom time. The climatic projections were based on two distinct scenarios: the first scenario involves a gradual reduction in emissions resulting in limited warming of 1.1 °C (SSP1-2.6) for both 2050 and 2100, while the second scenario assumes an unchanged emission pathway with a mean warming of 1.6 °C and 3.8 °C (SSP5-8.5)^13^ for 2050 and 2100, respectively. To estimate the average impact on the bloom DOY while accounting for yearly variability, we applied an average winter temperature increase to both the minimum and maximum daily temperatures (ΔT_min • max_) to each of four consecutive years (2019, 2020, 2021 and 2022). This time span exhibits inter-annual winter temperature variability while maintaining natural weather patterns. Additionally, following observations in Fig. 1a, we analyzed a rise only in daily maximum temperature (ΔT_max_) and kept the nighttime temperature constant. Generally, both SSP1-2.6 and SSP5-8.5 scenarios predicted a significant impact of future climate shifts on bloom time (ANOVA; F(6, 63) = 15.8; p < 0.0001). Post-hoc tests using the Dunnett method indicated that all tested scenarios would advance bloom’s DOY. Under the reduced emission scenario (SSP1-2.6) for 2050 and 2100, the modeled bloom DOY shifted from 64 (March 5th) to 52 (February 20th) in the ΔT_min • max_ model and to 51 by the ΔT_max_ scenario (p = 0.0055 and p = 0.0068, respectively). If emissions do not change (SSP5-8.5), the ΔT_min • max_ model projected that bloom DOY would advance to 48 (February 17th) by 2050, and the ΔT_max_ model predicted that almond trees would bloom by DOY 49 (February 18th). Predictions for 2100 suggest that trees would bloom by January 28th (DOY 28) or February 2nd (DOY 33) by the ΔT_min • max_ and ΔT_max_ scenarios, respectively (p < 0.0001; Fig. 3a).

**Figure 4:**
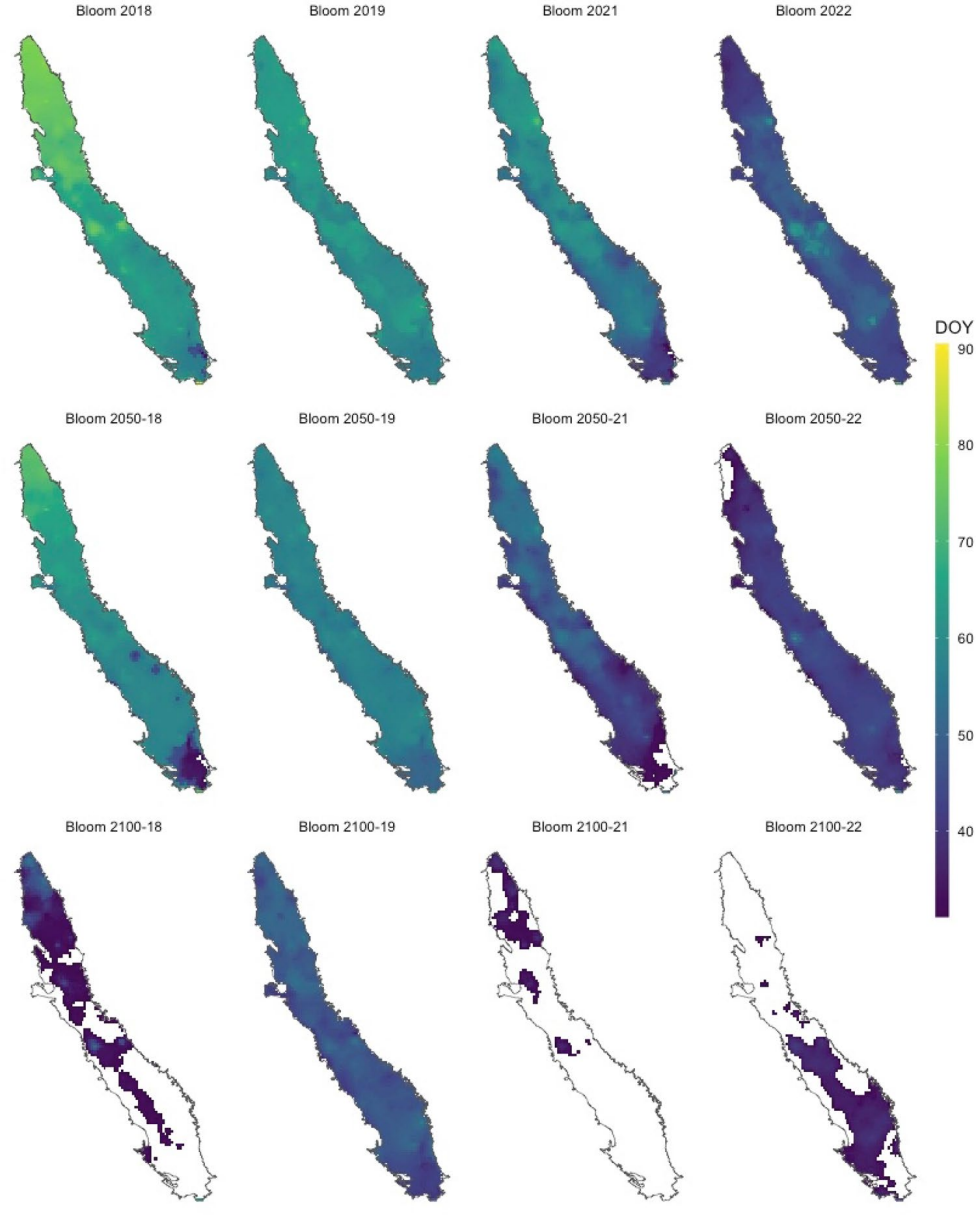
Projected spatial variability in almond bloom time. Bloom day of the year (DOY) between 32 (February 1st) and 90 (April 1st) across the California Central Valley. To account for year-to-year variability, we conducted four different simulations for 2050 and 2100 based on the winters of 2018, 2019, 2020 and 2022. The white area represents land area unsuitable for almond production.

To identify regions suitable for sustained almond production, we focused on bloom projections within two critical windows. We considered after February 1st (DOY 32) to minimize frost damage and low bee activity, and before March 31st (DOY 90) to lessen the likelihood of encountering high temperatures (> 21 °C), which can adversely impact pollen production. Based on these thresholds we determined that over the past 40 years (1982–2022), a substantial portion (43,416 km^2^) of Central Valley's total area (~ 47,000 km^2^) has been suitable for almond production. An ANOVA analysis exhibited a significant main effect of the future climatic scenarios on bloom area (F(6, 63) = 25.8; p < 0.0001). Using the past as a control and conducting posthoc testing with the Dunnett method, results suggest that there will be an insignificant reduction in the suitable land between 40,800–42,800 km^2^ (p > 0.8) by 2050. However, a significant decrease in suitable area is predicted for 2100 by the unchanged-emission scenario (SSP5-8.5), with large portions of Central Valley becoming unfit for almond production under both ΔT_min • max_ and ΔT_max_ scenarios, leaving ~ 11,700 km^2^ or ~ 22,200 km^2^ productive (respectively; p < 0.0001; Figs. 3b, 4).

## Discussion

The impact of climate change on tree phenology is one of the most discussed aspects of global warming^2,15,16^. Presumably, higher temperatures will result in earlier bloom. Indeed, our analysis suggests that over the last 40 years, the rate of change in the bloom of almond trees was 0.11 days year^−1^ resulting in a shift of 1.1 days over a decade. This rate of change is similar to several long-term observational studies showing that dormancy break shifted by a few days over the last few decades with a typical rate of 0.05–0.28 day year^−1^^2,17,18^. Our model, which links carbohydrate content in the fall with winter temperatures to predict bloom^10,11^, suggests that this trend will continue under the most optimistic CO_2_ emissions scenario (SSP1-2.5) over the next few decades and stabilize by the end of the century. However, supposing no emission reduction measures are implemented (SSP5-8.5), the trend towards earlier blooms may persist or even accelerate from approximately 1.1 days per decade (observed over the past 40 years) to nearly 2.5 days per decade under the ΔT_min • max_ model conditions. This substantial shift could lead to significant losses of prime orchard areas.

In California, almond trees generally bloom between early February and late March. This period aligns with a reduced likelihood of hard frost, typically occurring from mid-December to January, which could potentially harm or destroy the flower's ovules. Based on our analysis, we found that minimum temperatures have remained relatively stable over the past 40 years. This suggests that the threat of frost continuous to be a concern. However, it’s important to note that this may change in the future. According to future climate projections, the risk of frost damage is expected to decrease in the coming decades for almonds in the Central Valley^19,20^. While the risk is expected to decrease, it will not be completely eliminated in the future^21^. Within this context it is essential to recognize that the C-T model does not explicitly account of the risk of late frost damage thus we have set a threshold of February 1st (DOY 32) in consideration of when frost damage is more likely to occur. Additionally, excessively high temperatures (above 21 °C), can pose a significant barrier for almond pollen production^22^ thus March 31st (DOY 90) was established as an additional threshold. Using these two dates as thresholds for land suitability for almond production, our analysis suggests that California’s Central Valley currently provides adequate conditions for timely bloom. Moreover, it is projected that 95 percent of the area will continue to maintain favorable spring conditions for almond bloom for the next 30 years or even until the end of the century if greenhouse gas emission decrease (SSP1-2.6 scenario). However, if emissions continue to rise unchecked (SSP5-8.5), we expect a significant loss in arable lands for almond production. A 48% loss in almond production is predicted under the ΔT_max_ scenario, and approximately 73% loss under the ΔT_min • max_ scenario. Hence, despite comforting predictions that California will be climatically favorable for almond production for the next 30 years, it appears almond farming is not immune to climate shifts. If carbon emissions are not reduced, California will not be as suitable for almond production by 2100. Considering that almond orchards’ longevity is 25–30 years, we might be planting the last cohort of California’s almond farms.

Prior studies have indicated potential arable land losses due to insufficient water availability or changes in summer temperature^23^. Furthermore, research suggests that plant performance is more sensitive to variations in water availability or precipitation rather than extreme temperatures^24–26^. Since the majority of orchards in California's Central Valley are irrigated, any loss of farmland due to summer climate changes may not be directly attributed to temperature fluctuations, but rather to changes in water allocation or advancements in irrigation technology. Nevertheless, the impact of winter climate shifts on perennial plants is insufficiently understood by researchers or stakeholders. Changes in winter temperatures can influence the use of energy reserves (NSC) and trigger earlier blooms. These early blooms may expose plants to frost or create conditions that adversely affect pollination, thereby reducing crop potential. The loss of the most productive almond-growing area in the world, which accounts for nearly 80% of total global output, would be devastating, as similar Mediterranean-like climatic regions are scarce. Recognizing that the loss of arable lands may be inevitable, we can proactively allocate resources to develop techniques and mitigate the impact of winter temperatures on phenology. Focusing on innovative approaches and adaptive measures may preserve the productivity of these vital almond-growing regions and ensure the sustainability of almond production in the face of climate change.

## Data Availability

Available upon request from the corresponding author, Jessica Orozco.
